# PRDM15 interacts with DNA-PK-Ku complex to promote radioresistance in rectal cancer by facilitating DNA damage repair

**DOI:** 10.1038/s41419-022-05402-7

**Published:** 2022-11-19

**Authors:** Yue Yu, Tingting Liu, Guanyu Yu, Hang Wang, Zhipeng Du, Yuanyuan Chen, Nan Yang, Kun Cao, Chunlei Liu, Zhijie Wan, Hui Shen, Fu Gao, Yanyong Yang, Wei Zhang

**Affiliations:** 1 Department of Colorectal Surgery, Changhai Hospital, Naval Medical University, Shanghai, China; 2 Department of Radiation Medicine, Faculty of Naval Medicine, Naval Medical University, Shanghai, China; 3 School of Public Health and Management, Wenzhou Medical University, University Town, Wenzhou, Zhejiang China; 4 Pharmacy Department, Qingdao Special Servicemen Recuperation Center of CPLA Navy, Qingdao, 266071 China; 5 Chifeng Municipal Hospital, Chifeng Clinical Medical School of Inner Mongolia Medical University, Chifeng, 024000 China

**Keywords:** Preclinical research, Oncogenes

## Abstract

Neoadjuvant radiotherapy is a standard treatment for locally advanced rectal cancer, however, resistance to chemoradiotherapy is one of the main obstacles to improving treatment outcomes. The goal of this study was to explore the role of PRDM15 involved in the radioresistance of colorectal cancer and to clarify the underlying mechanism. In present study, we demonstrated that, after DNA damage, PRDM15 was upregulated and localized to DNA damage sites, co-localizing with γ-H2AX. Knockdown of PRDM15 inhibited DNA damage repair and increased radiosensitivity in colorectal cancer cells. Mechanistically, PRDM15 promoted DNA repair by interacting with DNA-PKcs and Ku70/Ku80 complex. In preclinical models of rectal cancer, knockdown of PRDM15 sensitized cell derived xenograft and patient derived xenograft to radiotherapy. In 80 rectal cancer patients treated with neoadjuvant chemoradiotherapy, higher PRDM15 expression was observed associated with weaker tumor regression and poorer prognosis. Our findings revealed that inhibiting PRDM15 was potent to overcome radioresistance through abrogating DNA repair in colorectal cancer cells. Additionally, the expression level of PRDM15 could be applied to predict radiotherapy responsiveness and the outcome of neoadjuvant radiotherapy in rectal cancer patients.

## Background

Colorectal cancer (CRC) has ranked third in the global cancer incidence and is the second leading cause of cancer-related deaths worldwide [1]. Currently, multidisciplinary therapy for locally advanced rectal cancer patients, such as neoadjuvant radiotherapy followed by operation has become the standard of therapy [2, 3]. Radiotherapy with or without chemotherapy has been widely used to improve outcomes, including local control and long-term survival [4, 5]. However, a subpopulation of cancer cells manifesting intrinsic radioresistance may survive such multidisciplinary treatment. Simultaneously, cancer cells also gradually developed a radioresistant phenotype during the radiotherapy was applied. In addition, reactivation of proliferation in these cells and successful colonization at local or distant organs can lead to local recurrence and distant metastasis, which may increase the risk of poor outcomes [6, 7]. Developing novel strategies to overcome radioresistance is of great importance for overall outcome of colorectal cancer patients.

The primary mechanism of cancer radiotherapy is to induce abundant DNA damage. However, the repair of damaged DNA in cancer cells determines radiosensitivity [8, 9]. As the most severe type of DNA damages, DNA double stranded breaks (DSB) resulting from radiotherapy in cancer cells often leads to cell apoptosis, autophagy as well as cell cycle arrest. However, cancer cells harbor stronger capacity to repair damaged DNA and may survive under radiotherapy, which promotes radioresistance. Several strategies have been developed to interrupt DSB repair pathway including Non-homologous end joining (NHEJ) and homologous recombination (HR) repair, the most two predominant DSB repair pathways [10]. Identifying novel factors involving DSB repair also provides possible target for radiosensitization [8, 9, 11].

PR-domain-containing proteins (PRDMs) are zinc-finger sequence-specific chromatin factors that play essential roles in embryonic development and cell fate decisions [12–14]. Among all PRDM members, PRDM15 was reported to regulate the naive state of mouse embryonic stem cells [15, 16]. Mechanistically, PRDM15 modulates WNT and MAPK–ERK signaling by directly upregulating Rspo1 and Spry1 [15]. PRDM15 promotes a transcription that maintains the activity of the PI3K/AKT/mTOR pathway and glycolysis in B-cell lymphomas [17]. Knockout of PRDM15 induces a metabolic crisis and selective death of lymphoma cells. In addition, PRDM15 regulates the transcription of pivotal factors of the NOTCH and WNT/PCP pathways to preserve early midline structures in the developing embryo [18]. Despite its characterized role in stem cell biology and during early development, the role of PRDM15 in cancer remains obscure.

In the present study, we reported that PRDM15 confers radioresistance of colorectal cancer through facilitating NHEJ repair machinery. Knockdown of PRDM15 in cancer cells or patient tissues derived xenograft increased the efficacy of radiotherapy. Furthermore, a higher expression of PRDM15 was found to be associated with poorer prognosis in locally advanced rectal cancer patients treated with neoadjuvant chemoradiotherapy. Our findings provide novel mechanism and target for rectal cancer radiotherapy.

## Materials and methods

### Cell lines and treatment

Human colorectal cancer cells HCT116, HT29, WiDr cells; and human renal epithelial 293T cells were obtained from American Type Culture Collection (ATCC) and cultured according to ATCC guidelines. Briefly, HCT116, HT-29 and WiDr cells were maintained in RPMI 1640 medium (HyClone) supplemented with 10% fetal bovine serum (FBS, Gibco). 293T cells were maintained in DMEM (Hyclone) with 10% FBS. In addition, 1% penicillin streptomycin-glutamine was added to the medium, and the cells were maintained at 37 °C in a humidified incubator containing 5% CO_2_. Cells were treated accordingly and used in next experiments.

### Lentivirus packaging and stable cell line construction

For lentivirus packaging, logarithmically growing 293T cells were plated in a 10 cm dish at a cell density of 1 × 10^7^. According to the instructions of the viral packaging kit, PRDM15-knockdown (PRDM15-KD, Supplementary Table 1) and PRDM15-overexpressing (PRDM15-OE, Supplementary Table 1) plasmids (Zorin BioTech., Shanghai, China) and viral plasmids were synthesized by Zorin Biotech. Co. (Shanghai, China) and transfected into 293T cells. Then, the supernatants of the sh-PRDM15 and PRDM15-OE cells were collected at 24, 48, and 72 h after transfection. The collected virus supernatants were centrifuged at 3500 rpm for 10 min and filtered through a filter of 0.22 μm. When the cell density of logarithmically growing cells reached 60–70%, we added the virus supernatant to HCT116 or HT29 cells. Polybrene (5 μg/mL) was added to the culture medium for 48–72 h. Puromycin (800 ng/mL) was added for selection, and the cells that survived for 96 hours were considered stable strains.

### Ionizing radiation (IR)

The ^60^Co γ-rays were applied for the radiation exposure. Cells were irradiated with 2, 4, 6 or 8 Gy at a dose rate of 1 Gy/min. Mice were subjected to local pelvic cavity radiotherapy with 15 Gy once.

### Inhibitors of kinases

Three key upstream kinases in the DDR pathway, DNA-PK, ATM and ATR, were selected. The inhibitors were NU7441, KU55933 and VE821, respectively, and their working concentrations were 10 μM. The inhibitor was applied to the cells 2 h before intervention, to ensure that they could exert the inhibitory effect on the target kinase in advance. Cells were treated with 8 Gy IR, washed with PBS for three times, and then cultured with complete medium containing inhibitors, respectively, until the fixed time point after IR.

### Clonogenic assay

Cells in the logarithmic growth period were digested and seeded in six-well plates at appropriate density. Cells were irradiated with different radiation dose of ^60^Co (0, 2, 4, and 6 Gy) with three replicates for each group. The cells were cultured for 12 days after IR. After washed with PBS, the cells were fixed with 4% paraformaldehyde and stained with crystal violet staining solution (Beyotime, China) for 20 min. Finally, the plates were gently washed with water and dried naturally. Colonies consisting over 50 cells were counted.

### Apoptosis assay

The Annexin V-APC/7-AAD Apoptosis Detection Kit (Yeasen, China) was used to explore cell apoptosis according to the manufacturer’s instructions. Briefly, the cell supernatant was collected 24 h after IR. The cells were digested with trypsin (without EDTA) and collected with the supernatant. The cells were centrifuged and washed three times with PBS. Then, the cells were resuspended in binding buffer and the Annexin V and 7-AAD were added according to the manufacturer’s instructions. The cells were incubated for 5 min in the dark at room temperature. Finally, the cells were analyzed by flow cytometry (Beckman, USA).

### Cell viability assay

Cell viability was detected by Cell Counting Kit-8 (CCK-8, Dojindo, Japan). Cells were suspended and seeded into 96-well plates with 3 × 10^3^ cells/well. At 24, 48, and 72 h after IR, cell viability was tested with a CCK-8 assay at OD450 nm.

### Cell cycle analysis

Cell cycle was assayed by PI staining. The cells were plated in six-well plates at a density of 2 × 10^6^ cells/well. The cells were digested at 0, 4, 8, 12, 24 h after IR, then washed with PBS twice, fixed with 75% ethanol for 24 h, and washed again twice with PBS. Finally, cells were stained with PI for 30 min and assayed by flow cytometer (Beckman, USA). We compared the difference in G2/M phase levels between PRDM15-NC and PRDM15-KD cells. All the experiments were performed for three independent times.

### Neutral comet assay

The comet assay was used to detect DSBs induced by radiation. Firstly, slides were immersed in 1% naturally molten agarose and dry thoroughly. Next, the prepared single cell suspension (2 × 10^4^ cells/mL) was immersed in low melting agarose in 40 °C water. Thirdly, cell suspension was mixed and rapidly pipetted onto the surface of the precoated slide. The slides were then incubated at 4 °C for 25 min at 25 V in TBE buffer (0.744 g Na_2_EDTA, 5.564 g boric acid, and 10.902 g Tris in 500 ml double distilled water, pH 8.2–8.5). Then the gel was stained with PI (10 µg/ml) for 20 min and then rinsed gently with ddH_2_O. Finally, all gels were examined by Olympus BX60 fluorescence microscope. Total 100 images in each slide were analyzed using CASP 1.2.3b2 software (CAS-Plab, Poland).

### Western blotting (WB)

After different treatments, proteins were extracted with M-PER Mammalian Protein Extraction Reagent (Thermo Fisher Scientific, Taiwan) according to the manufacturer’s instructions. Proteins were separated on a 10% sodium dodecyl sulfate-polyacrylamide gel electrophoresis gel (SDS-PAGE gel, Beyotime, China) and transferred to nitrocellulose membranes (Millipore, USA). Five percent skimmed milk was used to block the membranes for 1 h and then the membranes were incubated overnight at 4 °C with the primary antibodies listed in Supplementary Table 2. Next, the membranes were incubated with respective secondary antibodies (Proteintech, China) for 1 h at room temperature. Peroxidase labeling was visualized via enhanced chemiluminescence labeling using an ECL Western blotting detection system (Thermo Fisher Scientific, Waltham).

### Co-immunoprecipitation (Co-IP)

Co-immunoprecipitation were performed to determine the interaction between proteins after IR as previous described [19]. Briefly, for Co-IP, 8 h after HCT116 cells were irradiated at a dose of 8 Gy, proteins were extracted. Then, proteins were immunoprecipitated with PRDM15 or DNA-PKcs specific antibody using the Pierce™ Co-Immunoprecipitation Kit (Pierce, #26149) according to the manufacturer’s instructions. After immunoprecipitation, proteins were subjected to SDS-PAGE and immunoblotted with an antibody for DNA-PKcs, Ku70, Ku80 and PRDM15.

### Immunofluorescence staining (IF)

Cells seeded on 22 × 22 mm^2^ cover slips in six-well plates were irradiated and fixed at indicated time points by 4% paraformaldehyde for 30 min. Then, cells were permeabilized by 0.5% Triton X-100 buffer. After blocking in 1% BSA for 1 h at room temperature, the cells were incubated with γ-H2AX (Ser139) antibody (Abcam), PRDM15 (Abcam), DNA-PKcs (Abcam) or 53BP1 antibody (Abcam) at 4 °C overnight. Then, the cells were washed with PBS twice and incubated with FITC-labeled anti-mouse antibody (Abcam) or Texas Red-labeled anti-rabbit antibody (Abcam) at room temperature for 2 h. Nucleus was stained with DAPI for 15 min in the dark. Images were obtained by a confocal microscope (Zesis 880, USA) with the NIS-Elements Viewer 4.20 capture system. Six slices of each specimen were observed and five high-power visual fields were randomly selected. For protein co-localization analysis, the images of PRDM15 together with DNA-PKcs, Ku70 or Ku80 in 100 nuclei from three independent experiments were analyzed with Image J software. The Pearson correlation coefficient (PCC) and Overlap Coefficient (OC) are used as a statistic to quantify co-localization. The PCC and OC was measured using the Image J plugin Colocalization Finder [20]. The plugin can be downloaded from http://questpharma.u-strasbg.fr/html/colocalization-finder.html.

### Xenograft model and local radiotherapy

For cell derived xenograft (CDX) model, BALB/c-nude mice (male, 6 weeks old, obtained from the Experimental Animal Center of Naval Medical University) were kept in the Animal Room of the Department of Radiation Medicine, Naval Medical University. 5 × 10^6^ HCT116 cells (PRDM15-NC or PRDM15-KD) in the logarithmic growth phase were collected and subcutaneously injected into both thighs of each mouse. For patient-derived xenograft (PDX) model, tumor tissues from rectal cancer patients were implanted to nude mice and established by LIDE Biotech Co., Shanghai, China. Mice bearing passage 3 PDX model (COPF161282) were randomly injected with PRDM15 knockdown virus and divided into 4 groups: PRDM15-NC IR group, PRDM15-KD IR group, PRDM15-NC non-IR group and PRDM15-KD non-IR group. Tumor bearing Mice received a pelvic cavity local ^60^Co 15 Gy exposure, with the residual region of the body shielded with lead (Supplementary Fig. 1). Tumor volume was measured every 4 days until 28 days after IR, and then mice were euthanized. The tumor xenografts were excised, fixed, weighed, photographed, and stored. All the animal experiments were carried out with the approval of the Ethics Committee of Naval Medical University.

### IHC and TUNEL staining

When the CDX or PDX tumors reached 100 mm^3^, mice were subjected to local radiotherapy of 15 Gy, and the tumors were isolated at 24 h after IR or at the end point of observation for immunohistochemistry. After fixed in 4% paraformaldehyde for 24 h, the tumors were embedded in paraffin, and cut into 3 μm sections. Ki67 was immunohistochemically analyzed. Moreover, the slides were stained with TdT-mediated dUTP nick-end labeling (TUNEL) and observed under a microscope (Zesis, USA) equipped with NIS-Elements Viewer 4.20 capture system. Three slices of each specimen were observed, and five high-power visual fields were randomly selected from each slice. The positive cell percentage were analyzed by Image J software. The IHC Profiler plugin was used to automatically score the staining of the sample, and then, the Trainable Weka Segmentation plugin was used to count positive and negative cells.

### Patients samples and tissue microarray

80 locally advanced rectal cancer patients who had received neoadjuvant chemoradiotherapy from May 2016 to October 2019 at Department of Colorectal Surgery Shanghai Changhai Hospital, Naval Medical University (Shanghai, China) were included in this study. For inclusion, the patients were required to have only one primary lesion, have completed standard neoadjuvant chemoradiotherapy, have undergone surgical resection, and have survived at least 1 month after surgery. The study was approved by the Ethics Committee of Changhai Hospital. Tumor tissues and tissues adjacent to carcinoma were obtained after surgery and then paraffin-embedded into a microarray for IHC (Supplementary Table 3). Neoadjuvant chemoradiotherapy was performed by 3D conformal technique, conventionally fractional. The total dose was 50 Gy, 2 Gy per fraction. Capecitabine monotherapy regimen was selected for concurrent chemotherapy. Preoperative and postoperative chemotherapy cycle was conventionally 6 months in total. The interval between preoperative radiotherapy and surgery was generally 6–8 weeks. Sensitivity to neoadjuvant chemoradiotherapy was evaluated with the tumor regression grade (TRG). TRG 0 (complete regression): no visible tumor cells under microscope; TRG 1 (near complete regression): microscopy showed only single or small tumor cells. TRG 2 (partial regression): significant regression with more residual tumors than single or small tumor cells; TRG 3 (poor or no regression): residual tumor is extensive without significant regression.

### Clinical analysis for tissue microarray

IHC assays were conducted to detect PRDM15 protein levels in cancer tissues and tissues adjacent to carcinoma, after which PRDM15 levels were independently and semi-quantitatively assessed by two pathologists using the histochemistry score (H-score, ranging from 0 to 300). We evaluate the PRDM15 by the ratio of the H-score in cancer tissues to that in adjacent tissues. The cut-off value for PRDM15 level was deduced according a receiver operating characteristic (ROC) curve, and patients were categorized into two groups based on PRDM15 level (high or low).

### Statistical analysis

Data are expressed as the means ± standard deviation (SD) for each independent experiment. In general, one-way analysis of variance followed by the Newman–Keuls multiple comparison test were used to compare more than two groups. Student’s two-tailed unpaired *t* test was used to compare differences between two groups. *P* value of < 0.05 was considered to indicate a statistically significant result. GraphPad Prism 6 Software (GraphPad Software Inc., La Jolla, CA) was utilized for all statistical analyses and construction of graphs. All experiments were performed with at least three independent experiments.

## Results

### PRDM15 is responsive to radiation induced DNA damage in CRC cells

**Fig. 1:**
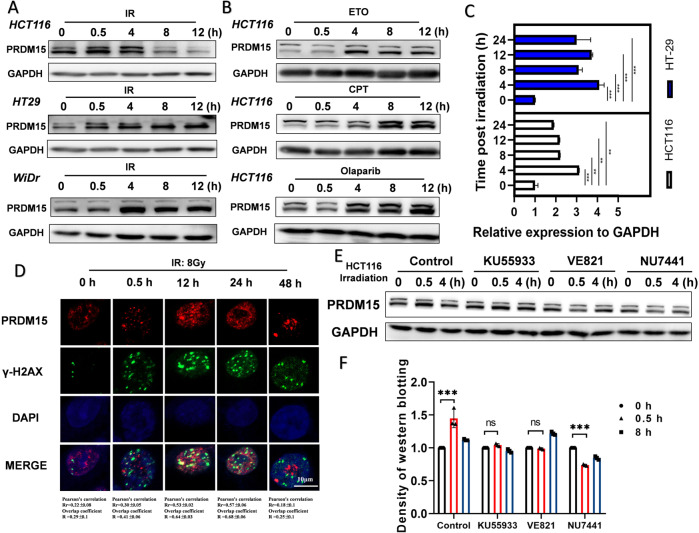
PRDM15 is responsive to DNA damage in colorectal cancer cells. **A** Representative images of Western Blot of PRDM15 expression in different colorectal cancer cells after 8 Gy IR at different time points. **B** Representative images of Western Blot of PRDM15 expression in HCT116 with chemotherapeutics at different time points. **C** Expression of PRDM15 mRNA in HCT116 and HT29 cells after IR. Error bars represent the SD of the mean of three independent experiments. Student’s *t* test was applied in those experiments after analyzing with ANOVA. *P < 0.05, **P < 0.01, ***P < 0.001 and ****P < 0.0001 versus negative control. **D** The γ-H2AX and PRDM15 foci in HCT116 at different time after 8 Gy IR was analyzed by immunofluorescence staining. Co-localization of PRDM15 with γ-H2AX was performed by Image J. Rr represents Pearson’s correlation coefficient and R represents Overlap coefficient. Pearson correlation coefficient of 0.5–1 is considered as co-localization. (E) Representative images of Western Blot of PRDM15 expression at different time after IR with three different kinase inhibitors. Ku55933 for ATM inhibitor; VE821 for ATR inhibitor; Nu7441 for DNA-PK inhibitor. **F** Quantification of PRDM15 expression level with the three kinase inhibitors. *n* = 3 for each group per time point. Values are given as mean ± SD. *P < 0.05, **P < 0.01, ***<0.001 and ****P < 0.0001 versus untreated control group.

Firstly, expression of PRDM15 in different CRC cell lines were detected (Supplementary Fig. 2A). To determine whether PRDM15 plays a role in DNA damage response, we firstly determined its expression and localization in response to IR and other types of DNA damage. The results showed that at 0.5 h after 8 Gy IR, the expression level of PRDM15 in cancer cells increased markedly at 0.5 h after IR and was decreased at 12 h (Fig. 1A, Supplementary Fig. 2B). Then we measured the expression of PRDM15 after Etoposide (ETO) or Camptothecin (CPT) treatment and found that the expression level of PRDM15 was significantly upregulated upon DNA damage (Fig. 1B, Supplementary Fig. 2B). The expression of mRNA level was also confirmed upregulated in both HCT116 and HT29 cells in response to IR (Fig. 1C). After known the expression of PRDM15 in response to DNA damage, we performed immunofluorescence staining to explore its localization at subcellular level. Surprisingly, PRDM15 showed a significant increase in the number of foci after 8 Gy IR (Fig. 1D). At 12 h and 24 h after IR, the PRDM15 foci was further observed to co-localize with γ-H2AX foci, an established marker of DSB, suggesting that PRDM15 was recruited to DSB sites and may play a direct role in DNA damage response (Fig. 1D). Then we determined the upstream DNA damage responsive kinase accounting for the upregulation PRDM15, and found that in DNA-PKcs inhibited cells, the increase of PRDM15 was abrogated (Fig. 1E, F). These data suggest that PRDM15 is responsive and related to radiation induced DNA damage.

### PRDM15 confers radioresistance in colorectal cancer cells

**Fig. 2:**
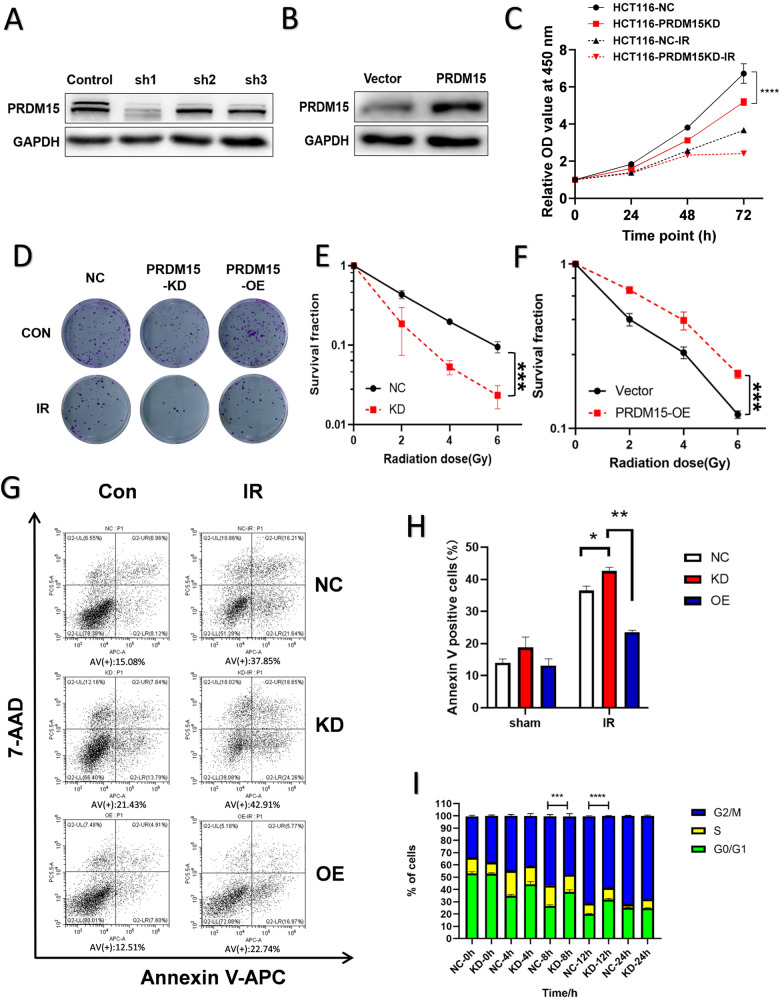
Identification of PRDM15 as a radioresistant gene in CRC. **A**, **B** The level of PRDM15 expression shown by Western Blot in HCT116 infected with PRDM15-specific shRNA or PRDM15 overexpression plasmids. **C** Cell proliferation of HCT116 in different groups after 8 Gy IR determined by CCK-8 assay. Cells transfected with shNC served as controls. Error bars represent the SD of the mean of three independent experiments. Two tailed Student’s *t* test was applied in NC and KD groups, respectively, after analyzing with ANOVA. *P < 0.05, **P < 0.01, ***P < 0.001 and ****P < 0.0001 versus untreated control. **D**–**F** Cell survival and its representative images of HCT116 in different groups after 0, 2, 4, 6 Gy IR. Colony analysis was performed at 12 days after IR. Cells transfected with shNC served as controls. Error bars represent the SD of the mean of three independent experiments, two tailed Student’s *t* test, *P < 0.05, **P < 0.01, ***P < 0.001 and ****P < 0.0001 versus untreated control. **G**, **H** The apoptosis of HCT116 after 8 Gy IR at 24 h in different groups was analyzed by flow cytometry. AV means Annexin V. Cells transfected with shNC served as controls. Error bars represent the SD of the mean of three independent experiments. One-way ANOVA was performed in the three groups, *P < 0.05, **P < 0.01. **I** The cell cycle of HCT116 after 8 Gy IR at 0,4,8,12,24 h in different groups was analyzed by flow cytometry, *n* = 6 for each group per time point. Values are given as mean ± SD, two-tailed Student’s *t* test, *P < 0.05, **P < 0.01, ***P < 0.001 and ****P < 0.0001 versus untreated control.

The upregulation and the formation of PRDM15 foci suggest possible roles in response to radiotherapy. To explore the role of PRDM15 in radioresistance, we stably knocked down and overexpressed PRDM15 in HCT116 cells (Fig. 2A, B). Among all the three shRNAs targeting PRDM15, PRDM15 ShRNA1 (shPRDM15-1) was able to knockdown both two bands of PRDM15. Therefore, we applied shPRDM15-1 to the subsequent assays. We found that PRDM15-KD cells showed reduced cell proliferation. The proliferation of PRDM15-KD cells also decreased after IR, although there was no statistical difference in this result (Fig. 2C). The clonogenic assays revealed that knocking down PRDM15 significantly reduced colony formation in HCT116 cells after IR, whereas overexpressing PRDM15 significantly enhanced colony formation in HCT116 cells after IR (Fig. 2D–F). Furthermore, cell apoptosis assay was detected with flow cytometry assay. After 8 Gy IR, we found the apoptotic cells in PRDM15-KD cells (24.26% + 18.65%) was significantly more than that in PRDM15-NC group (21.64% + 16.21%), while the proportion of apoptotic cells in PRDM15-OE group (16.97% + 5.77%) was significantly decreased (Fig. 2G, H).

Cells are largely arrested in the G2/M phase to repair the DNA damage caused by IR. In this research, HCT116 cells were arrested in G2/M phase and the level of G2/M cells peaked at 12 h after 8 Gy radiation. G2/M phase arrest was more severe in PRDM15-NC cells than in PRDM15-KD cells (Fig. 2I; Supplementary Fig. 3). It suggests that the cell cycle checkpoint activation may be abnormal in PRDM15-KD cells. Collective data above revealed that PRDM15 is involved in radioresistance of CRC cells.

### PRDM15 was required for efficient DNA damage response

**Fig. 3:**
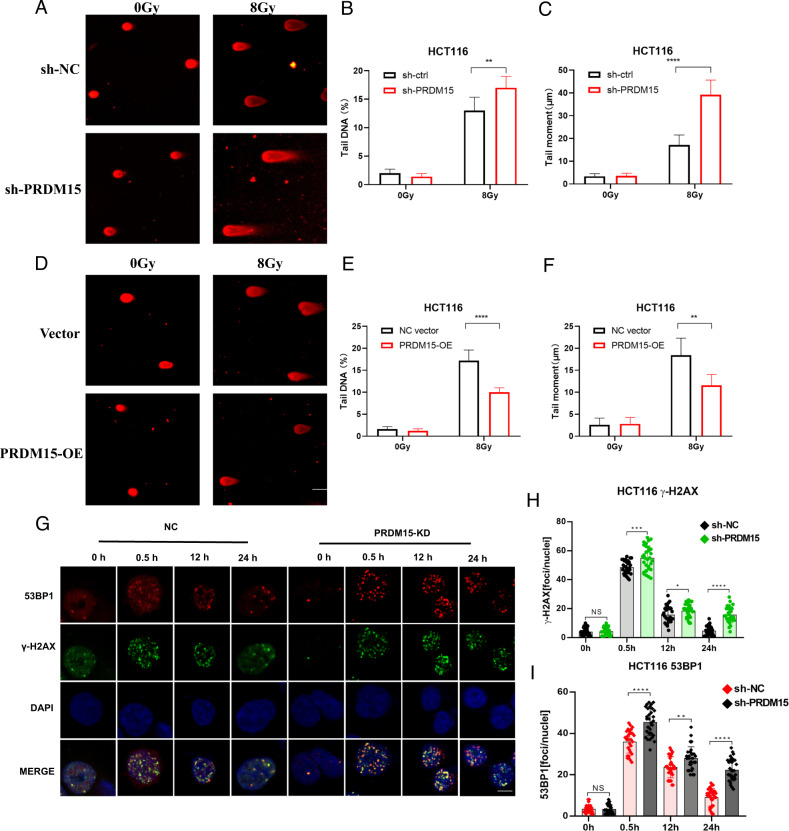
PRDM15 affects the process of radiation-induced DNA damage response. **A** Representative images from the comet assay of PRDM15-KD and NC cells at 8 h after 8 Gy IR. The tail DNA percentage (**B**) and tail moment (**C**) were quantified with CASP 1.2.3b2 software in PRDM15-KD and NC cells. Error bars represent the SD of the mean of three independent experiments, two tailed Student’s *t* test. *P < 0.05, **P < 0.01, ***P < 0.001 and ****P < 0.0001 versus shNC group. **D** Representative images from the comet assay of PRDM15-OE and control cells at 8 h after 8 Gy IR. The tail DNA percentage (**E**) and tail moment (**F**) were quantified with CASP 1.2.3b2 software in PRDM15-OE and NC cells. Error bars represent the SD of the mean of three independent experiments, two tailed Student’s *t* test. *P < 0.05, **P < 0.01, ***P < 0.001 and ****P < 0.0001 versus shNC group. **G** The γ-H2AX and 53BP1 foci in HCT116 after 8 Gy IR in different groups was analyzed by immunofluorescence staining. **H**, **I** Quantitative analysis of the number of γ-H2AX and 53BP1 foci per cell in different groups, *n* = 30 for each group per time point. Values are given as mean ± SD. *P < 0.05, **P < 0.01, ***P < 0.001 and ****P < 0.0001 versus shNC group.

The essential role of PRDM15 in radioresistance and its recruitment to DNA damage site promoted us to investigate its role in DNA damage repair. Then, we performed the comet assay. In the comet assay, more DNA damage was observed at 8 h in irradiated PRDM15-KD cells than that in NC cells (Fig. 3A–C). In contrast, less DNA damage was observed in PRDM15-OE cells after IR (Fig. 3D–F). These results suggested that loss of PRDM15 resulted in unrepaired DNA damage after IR. Through γ-H2AX foci assays, it was found that PRDM15-KD group showed more γ-H2AX foci unresolved than PRDM15-NC group, and were observed present until 24 h after IR (Fig. 3G, H). In addition, more 53BP1 foci were observed in PRDM15-KD cells than that in PRDM15-NC cells until 24 h after IR (Fig. 3G, I). These data indicate that PRDM15-KD cells exerted impaired capacity of DSBs repair.

**Fig. 4:**
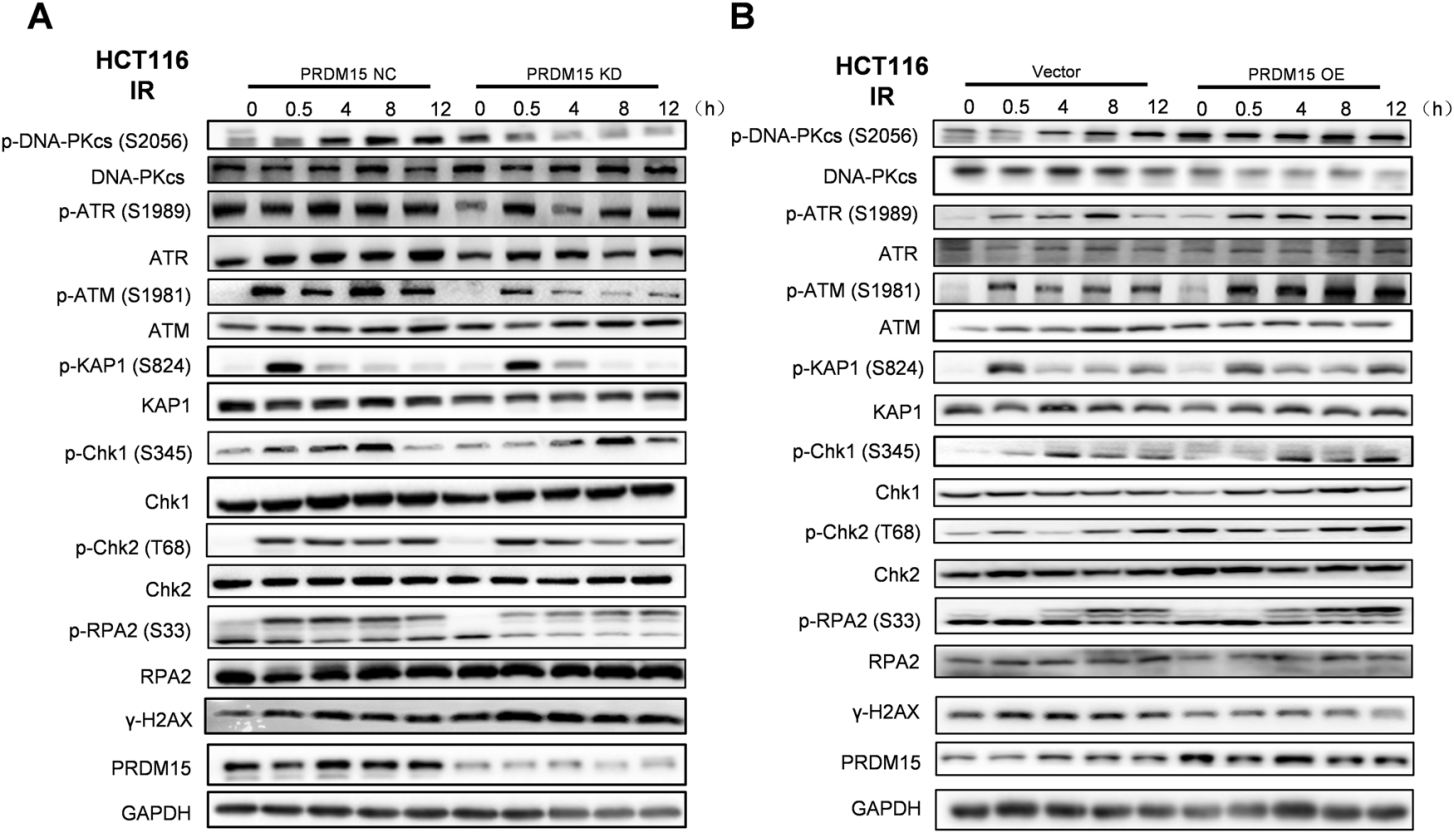
PRDM15 is required for the activation of DNA-PKcs mediated DNA repair. **A** Representative images of Western Blot of the protein expression in DNA Damage Response pathway in PRDM15-NC and PRDM15-KD cells after 8 Gy IR at different time points. **B** Representative images of Western Blot of the protein expression in DNA Damage Response pathway in PRDM15-NC and PRDM15-OE cells after 8 Gy IR at different time points.

To explore whether PRDM15 plays a role in the DNA damage response (DDR), proteins of cells in each group were extracted at 0, 0.5, 4, 8, and 12 hours after IR. It is known that activation of DDR is induced by phosphorylation of DNA-PKcs, ATM and ATR, as well as their substrates. However, in PRDM15-KD cells, the phosphorylation of DNA-PKcs at S2056 and the phosphorylation of ATM at S1981 were greatly decreased after radiation induced DNA damage (Fig. 4A; Supplementary Fig. 4A). And the overexpression of PRDM15 augmented the activation of DNA-PKcs and ATM signaling pathway (Fig. 4B; Supplementary Fig. 4B). In summary, it could be concluded that PRDM15 is highly likely to participate in NHEJ pathway through regulating DNA-PKcs.

### PRDM15 regulates DNA damage response by interacting with DNA-PK-Ku complex

**Fig. 5:**
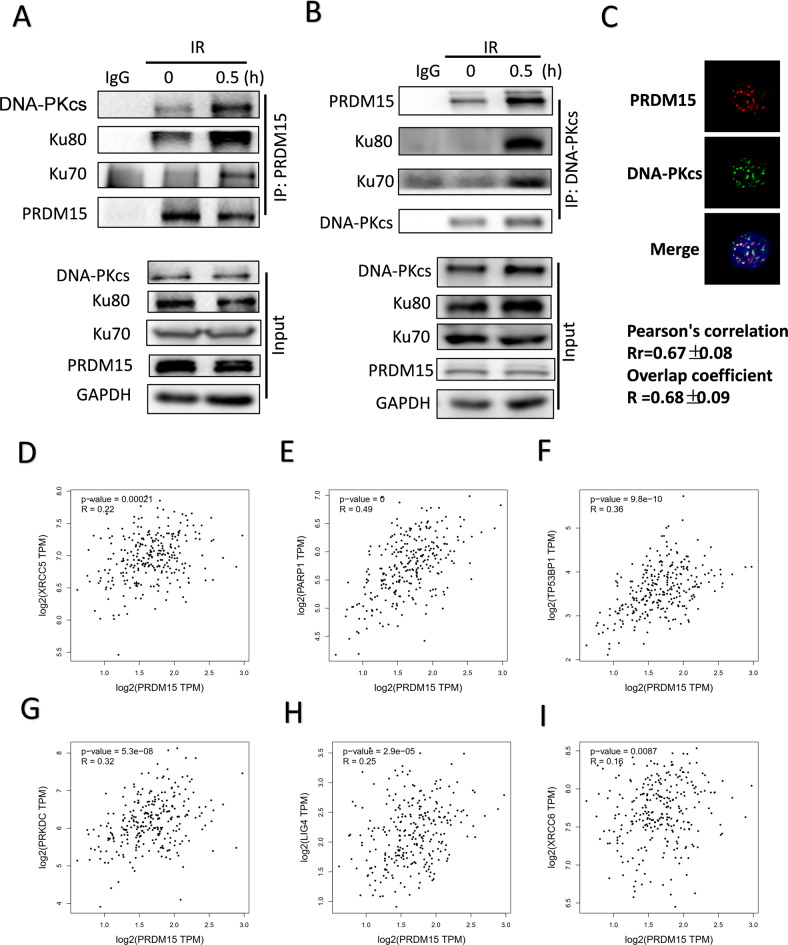
PRDM15 regulates NHEJ repair by interacting with Ku70/80. **A** Ku70, Ku80, and DNA-PKcs were immunoprecipitated by PRDM15 in lysates of irradiated HCT116 cells. **B** PRDM15 was immunoprecipitated by DNA-PKcs in lysates of irradiated HCT116 cells. **C** The representative IF images and quantification of co-localization of PRDM15 with DNA-PKcs. HCT116 cells at 0.5 h after IR were used for immunofluorescence staining. Confocal microscopy was applied to capture co-localization, and Image J was used to analyze the statistical significance of co-localization. Rr represents Pearson’s correlation coefficient and R represents Overlap coefficient. Pearson correlation coefficient of 0.5-1 is considered as co-localization. **D**–**I** Bioinformatics analysis of the correlation between PRDM15 and genes related to NHEJ pathway.

The impairment of DNA damage response in PRDM15-KD cells promoted us to investigate its direct target in response to DNA damage repair. Then, we performed Co-IP experiments and examined the key factors of DDR in proteins immunoprecipitated with PRDM15 antibody. Our data revealed that DNA-PKcs, Ku70, and Ku80 were immunoprecipitated by PRDM15 specific antibody in lysates of irradiated HCT116 cells (Fig. 5A). In order to compare the interaction of DNA-PKcs after IR more accurately, we adjusted the amount of PRDM15 to equalize its expression in Input. Then we found that the binding of DNA-PKCs, Ku70 and Ku80 to PRDM15 increased significantly at 0.5 h after IR (Fig. 5A).

Consistently, PRDM15 was also observed from the Co-IP lysates with DNA-PKcs antibody (Fig. 5B). The co-localization of PRDM15 and DNA-PK-Ku complex was also observed with confocal microscope after IR (Fig. 5C, Supplementary Fig. 5). The data above showed that DNA-PKcs may be a direct target of PRDM15. DNA-PKcs regulates DNA damage response mainly through phosphorylating substrates and promotes NHEJ pathway.

Then we performed bioinformatics analysis. Pearson correlation was applied in TCGA database to analyze PRDM15 and genes related to NHEJ pathway. We observed significantly correlation between PRDM15 and the key NHEJ genes, including, XRCC5 (Ku70), PARP1, T53BP1, PRKDC (DNA-PKcs), LIG4 as well as XRCC6 (Ku80) (Fig. 5D–I). Taken together, our data indicate that PRDM15 interacts with Ku70-Ku80-DNAPKcs complex to promote the repair of DSBs in CRC cells.

### PRDM15 promotes the radioresistance of rectal cancer in CDX and PDX model

**Fig. 6:**
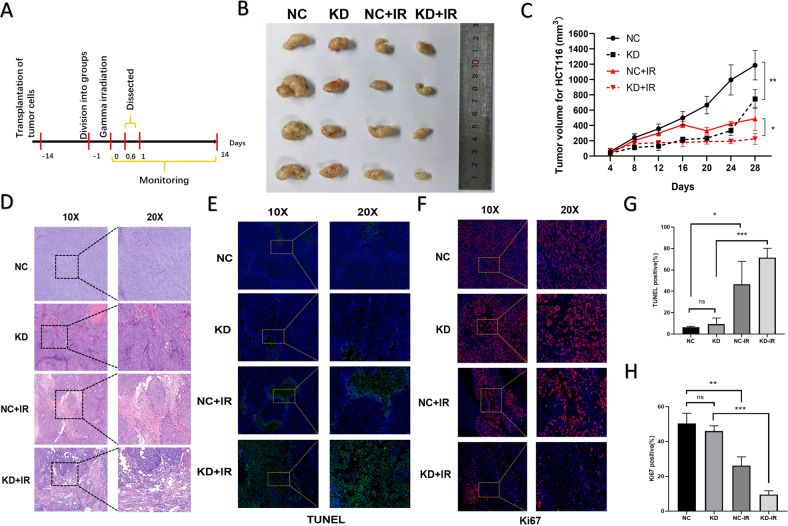
PRDM15 promotes the radioresistance of CRC in CDX models. **A** The overall design of the animal experiments. **B** Representative images of xenograft tumors in different groups at 28 days after 15 Gy local radiotherapy in pelvic cavity. **C** Tumor volume growth from the PRDM15-NC and PRDM5-KD groups with/without IR. Error bars represent the SD of the mean of four independent animals. two tailed Student’s *t* test was applied in NC and KD groups with the same intervention. *P < 0.05, **P < 0.01, ***P < 0.001 and ****P < 0.0001 versus untreated control. **D** Representative 10× and 20× images of H&E immunohistochemical staining of tumor tissue sections in different groups at 12 h after 15 Gy local radiotherapy in pelvic cavity. **E**, **F** Representative 20× and 40× images of TUNEL and Ki67 immunofluorescence staining of tumor tissue sections in different groups at 12 h after 15 Gy local radiotherapy in pelvic cavity. **G**, **H** Respective percentage of TUNEL (+) and Ki67 (+) cells in tumor by quantitative analysis. Data are shown as the mean ± SD, one-way ANOVA, *P < 0.05, **P < 0.01 and ***P < 0.001.

In order to explore the in vivo effect of PRDM15 on radiotherapy, we established subcutaneous CRC xenografts on nude mouse models with HCT116 cells. The mice were subjected to local radiotherapy at a single dose of 15 Gy to the abdomen after burdening HCT116 cells (Fig. 6A). After local radiotherapy, tumor sizes were measured every four days until 28 days, when the tumors were isolated for further analysis. After radiotherapy, the xenografts originated from PRDM15-NC HCT116 cells were larger than those originated from tumors derived from PRDM15-KD HCT116 cells (Fig. 6B). Furthermore, tumor growth was most rapid in the PRDM15-NC group without any intervention, and most inhibited in the PRDM15-KD group after IR. The PRDM15-KD group eventually grew faster than the PRDM15-NC plus IR group, possibly because the radiation had more profound tumor inhibition (Fig. 6C). By H&E staining, irradiated CDX model had larger areas of necrotic cells (visible neutrophil infiltration) than those in the unirradiated model. Compared with the PRDM15-NC model, tumors derived from PRDM15-KD cells showed more severe necrosis after IR (Fig. 6D). This suggests that knocking down PRDM15 can increase the degree of tumor damage after IR. TUNEL staining and Ki67 staining were performed to detect the cell apoptosis and proliferation rate of tumor cells. It was found that in the PRDM15-KD group, radiation-induced apoptosis of tumor cells was greatly increased (Fig. 6E, G). At the same time, the proliferation rate of tumor cells in PRDM15-KD group was significantly reduced than that in NC group, which indicates the knockdown group suffered more radiation damage (Fig. 6F, H).

**Fig. 7:**
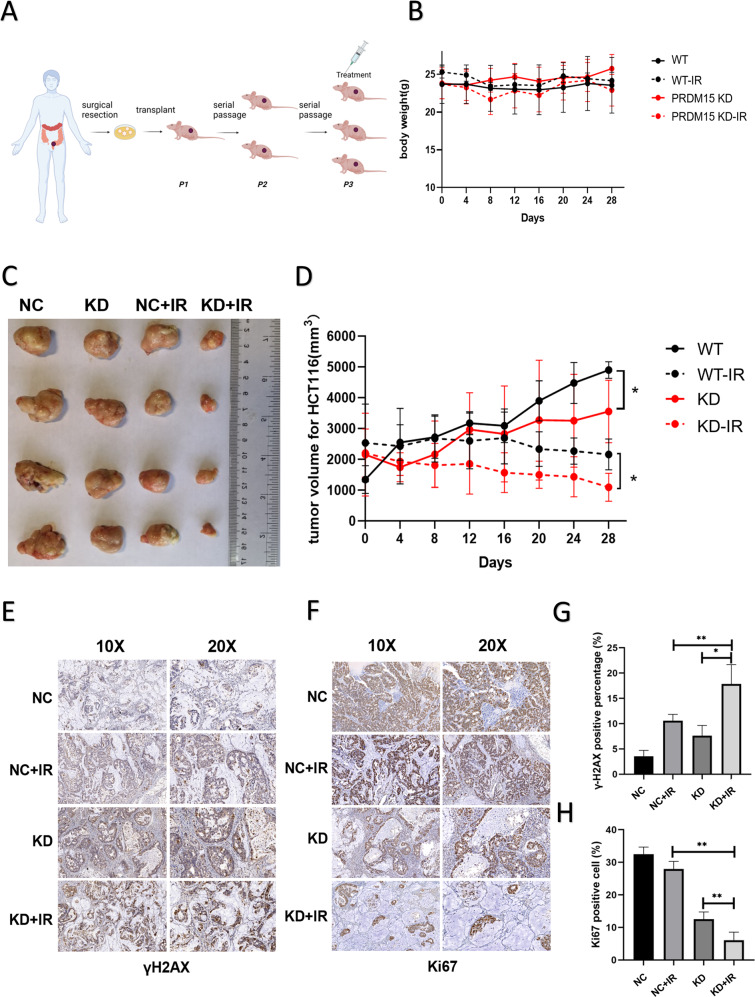
PRDM15 promotes the radioresistance of CRC in PDX models. **A** Schematic diagram of PDX model construction. **B** Mice weight monitoring of PDX models after radiotherapy. **C** Representative images of xenograft tumors in different groups at 28 days after 15 Gy local radiotherapy in pelvic cavity. **D** Tumor volume growth from the PRDM15-NC and PRDM5-KD groups with/without IR. Error bars represent the SD of the mean of four independent animals. two tailed Student’s *t* test was applied in NC and KD groups with the same intervention. *P < 0.05, **P < 0.01, ***P < 0.001 and ****P < 0.0001 versus untreated control. **E**, **F** Representative 10× and 20× images of γ-H2AX and Ki67 immunohistochemical staining of tumor tissue sections in different groups at 12 h after 15 Gy local radiotherapy in pelvic cavity. **G**, **H** Respective percentage of γ-H2AX (+) and Ki67 (+) cells in tumor by quantitative analysis. Data are shown as the mean ± SD, one-way ANOVA, *P < 0.05, **P < 0.01 and ***P < 0.001.

The data above suggested PRDM15 as a potent target to overcome radioresistance in CRC patients. Then the potential role of PRDM15 after IR was evaluated in the mice bearing subcutaneous PDX model (COPF161282). Mice bearing passage 3 PDX model were randomly divided into four groups, PRDM15-NC IR group, PRDM15-KD IR group, PRDM15-NC non-IR group and PRDM15-KD non-IR group, with four mice in each group (Fig. 7A). After the establishment of PDX model, lentivirus carrying PRDM15-NC and PRDM15-KD was injected into subcutaneous tumors. At 48 h after injection, the tumor region received a local radiotherapy at the dose of 15 Gy exposure. Tumor volume was measured every 4 days. The body weight of mice in each group were unaffected (Fig. 7B). As shown in Fig. 7C, D, tumor growth was most rapid in the PRDM15-NC group without any intervention, and most inhibited in the PRDM15-KD group plus IR. Similar to the data of CDX, PRDM15-KD group eventually grew slower than the irradiated PRDM15-NC group, largely due to the more profound effect of radiation on tumor suppression. After IHC staining for the DNA damage and cell proliferation, more unrepaired DSBs and less Ki67 positive cells were observed in PRDM15-KD plus radiotherapy groups than the single radiation group (Fig. 7E–H). These in vivo experiments confirmed that PRDM15 could be a potent target of radiosensitization in rectal cancer.

### High PRDM15 levels are associated with radioresistance and poor prognosis in locally advanced rectal cancer patients treated with neoadjuvant chemoradiotherapy

**Fig. 8:**
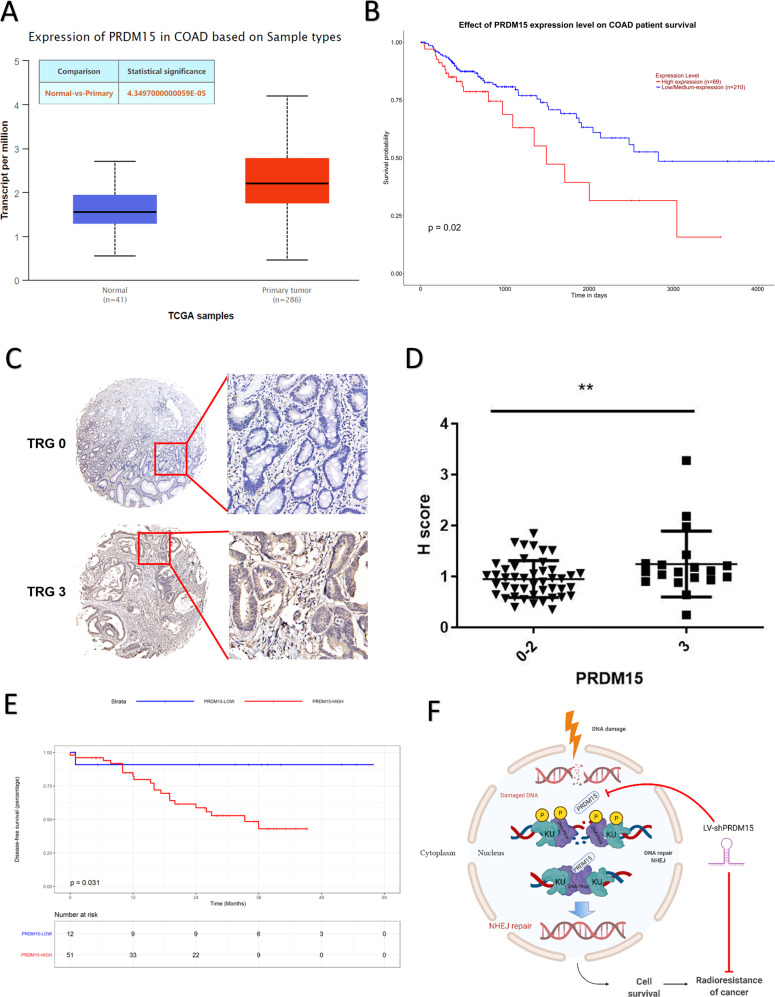
PRDM15 promotes radioresistance and poor prognosis in clinical patients. **A** The expression of PRDM15 in colon adenocarcinoma (COAD) (*n* = 41 for normal tissue, *n* = 286 for colorectal adenocarcinoma tissue, *p* < 0.05) in TCGA datasets. **B** Effect of PRDM15 expression level on colorectal adenocarcinoma patients survival. (*n* = 69 for high expression, *n* = 210 for low/medium-expression). **C** Tissue microarray results of PRDM15 expression in clinical rectal cancer. TRG: tumor regression grade. TRG = 0 indicates complete tumor regression; TRG = 3 indicates the worst tumor regression. **D** Immunohistochemical staining H score of PRDM15 in rectal cancer tissues. Cancer tissues from TRG3 group was considerably higher than that from TRG0-2 group. Values are given as mean ± SD, two tailed Student’s *t* test. **P < 0.01. **E** Kaplan–Meier survival analysis of clinical patients from the microarray. (F) Schematic diagram of how the PRDM15 regulates NHEJ repair and radiosensitivity.

The critical roles of PRDM15 in DDR and radioresistance provide great potential for clinical application. Bioinformatical analysis from the TCGA database also showed that PRDM15 was associated with colorectal cancer. PRDM15 expression in colon adenocarcinoma was higher than that in normal tissues (Fig. 8A). PRDM15 was also found to be negatively correlated with overall survival in CRC patients, suggesting PRDM15 as a factor of poor prognosis (Fig. 8B).

Then we detected its clinical relevance through using clinical samples collected in our department. Firstly, 80 patients who received neoadjuvant radiotherapy were included in our study, and patients were divided into a radiosensitive group (TRG 0-2) and radioresistant group (TRG 3) based on tumor regression grade (TRG). Then IHC staining of PRDM15 was performed in a tissue microarray consisting of all 80 patients (Supplementary Fig. 5; Table 4). We found that the expression level of PRDM15 in TRG3 cancer tissues was significantly higher than that in tissues from TRG 0-2 group (Fig. 8C, D; Supplementary Table 4).

Furthermore, we examined whether PRDM15 was associated with poor prognosis of patients. We obtained a cut-off value of PRDM15 level in CRC tissues with ROC cruve. This value optimally classified these 80 patients into two groups with high or low PRDM15 level. Kaplan–Meier survival analysis indicated that patients with high PRDM15 level had an inferior disease-free survival (DFS, *p* = 0.031, Fig. 8E).

These findings suggest that PRDM15 potentially confers radioresistance and poor prognosis in rectal cancer; thus, a high PRDM15 level might predict inferior tumor regression and prognosis in locally advanced rectal cancer patients receiving preoperative radiotherapy. And targeting PRDM15 could be a potential strategy for improving the radiotherapy effects in radioresistant rectal cancer patients.

## Discussion

Colorectal cancer is one of the most common malignant tumors and its morbidity and mortality are increasing yearly. Currently, neoadjuvant chemoradiotherapy reduces the rates of local progression in locally advanced rectal cancer [2]. As reported in NEJM by Rolf Sauer *et al*., the advantage of preoperative radiotherapy in reducing local recurrence rates was validated in a randomized controlled trial [21]. However, resistance to radiotherapy is the main cause of treatment failure or relapse in CRC. In the present research, we identified a novel factor PRDM15 is involved in DNA damage response and radioresistance of CRC.

To our knowledge, this is the first study demonstrating the role of PRDM15 in DNA damage and radiosensitivity of rectal cancer through regulating NHEJ mediated DNA damage repair. PRDM family members are sequence specific transcriptional regulators involved in cell identity and fate determination, often dysregulated in cancer [15–18, 22–30]. However, there is no evidence showing any correlation between PRDM15 and radiotherapy or DNA damage repair.

Our data showed that PRDM15 is responsive to IR as well as other types of DNA damage. Knocking down of PRDM15 significantly impaired DDR and increased radiosensitivity in CRC cells, CDX models and PDX models. Mechanistically, PRDM15 was found to directly interact with Ku70/80 complex to facilitate NHEJ repair of DNA damage. Furthermore, PRDM15 expression was also found to be correlated with patient survival in radioresistant and radiosensitive patients.

It is well known that radiation induces irreversible DSBs to kill tumor cells and that intensive repair of DSBs results in radioresistance in cancer cells [31, 32]. In this study, we confirmed that knockdown of PRDM15 promotes DNA damage in colorectal cancer cells and induces apoptosis. I advocate that we could further detect classical molecules in apoptosis pathway by Western Blot, for example, anti-apoptotic protein Bcl-2, apoptotic protein BAX and c-caspases3 (Cleaved caspases3). The expected results were that the expression of Bcl-2 was decreased in PRDM15-KD cells after IR, while the expression of c-Caspases3 and BAX was increased. In terms of cell cycle results, Richard Woo et al. demonstrated that DNA-PK activates p53 during DNA damage, enabling p53 to bind to specific DNA sequences [33]. Since phosphorylation of p53 by DNA-PK prevents MDM2 from degrading P53 [34], DNA-PK appears to serve a dual purpose: it activates DNA binding of p53 while blocking its inactivation by MDM2. DNA-PK mediates NHEJ, an imprecise form of DNA repair, which can be performed at any phase of the cell cycle. NHEJ repair only connects the two ends of DSB and has no potential to recover the original DNA sequence, making it a fast but error-prone repair method [35, 36]. In our following mechanism study, we found that PRDM15 was correlated with DNA-PK to some extent and played a role in NHEJ pathway. PRDM15 is likely to activate P53 to bind to DNA through DNA-PK and promote cell cycle retention in G2/M for DNA repair. However, after receiving IR, PRDM15-KD cells lacked the effect of PRDM15 and DNA-PK, and could not further activate the downstream effect of P53, so G2/M phase arrest of cells was inhibited. We could conduct further experiments on this hypothesis to confirm that PRDM15 does regulate P53 through mediating NHEJ pathway [37, 38].

In recent years, the treatment level of colorectal cancer continues to improve, and new chemotherapy drugs and targeted drugs continue to emerge, but more than 95% of the new drugs have been proved to be ineffective in human body in phase III clinical trials [39]. An important factor affecting the results of phase III clinical trials is the lack of preclinical models that accurately reflect drug efficacy. General animal models can’t simulate the function of matrix and immune components in human tumor microenvironment and tumor heterogeneity. However, these components play an important role in tumor resistance, invasion, metastasis and recurrence. With the continuous development of biotechnology, complex models including carcinogenic induction model, genetically engineered mouse model and PDX model have emerged, which can make up for the above deficiencies [40–42]. In this study, we firstly used CDX model, and found that tumors derived from PRDM15-KD cells are more sensitive to radiotherapy than tumor derived from PRDM15-NC cells. However, neither the subcutaneous model nor in situ rectal cancer model can reflect the real environment. We then generated the PDX model, and we injected the PRDM15-KD and PRDM15-NC lentivirus directly into the cancer tissues. These findings provide evidence that PRDM15 is a potential therapeutic target in terms of PDX model.

In addition, we found that the effect of PRDM15 on DNA damage repair was more obvious under the action of CPT. Moreover, CPT is commonly used in clinical treatment in rectal cancer [43, 44], which further proves that PRDM15 is associated with poor clinical prognosis, and also confirms that PRDM15 could also be a potential target for the combined treatment with CPT.

Our preclinical investigation revealed that high PRDM15 expression in cancer was significantly associated with poor tumor regression after neoadjuvant chemoradiotherapy; Moreover, PRDM15 upregulation predicted an inferior prognosis in locally advanced rectal cancer patients, who had received neoadjuvant chemoradiotherapy. Indeed, locally advanced rectal cancer patients with lower PRDM15 expression were more likely to acquire better tumor regression and survival outcomes. However, the exact role of PRDM15 in DNA damage response and cancer therapy remains to be investigated further.

## Conclusion

PRDM15 acts as a resistance factor that protects CRC cells from DSBs in vitro as well as in PDX models. Mechanistically, PRDM15 promoted NHEJ by interacting with DNA-PK complex. Moreover, PRDM15 upregulation was associated with inferior tumor regression and poor prognosis in locally advanced rectal cancer patients treated with neoadjuvant chemoradiotherapy. Therefore, PRDM15 might serve as an effective predictive biomarker of radiosensitivity and a target for radiosensitization in locally advanced rectal cancer patients; these findings may contribute to the precise treatment strategies in clinical practice in the future (Fig. 8F).

## Supplementary information

Supplementary figures and tables

Original Data File

## Data Availability

The datasets used and analyzed during the current study are available within the manuscript and its additional files.
