# Supplementary material for: PRDM15 interacts with DNA-PK-Ku complex to promote radioresistance in rectal cancer by facilitating DNA damage repair
Source: Cell Death Dis. 2022 Nov 19;13(11):978. doi: 10.1038/s41419-022-05402-7 (PMC9675803; doi:10.1038/s41419-022-05402-7)
Supplement: Supplementary file 1 — Supplementary figures and tables [file 41419_2022_5402_MOESM1_ESM.docx]

**Supplementary figures and tables for**

**PRDM15 interacts with DNA-PK-Ku complex to promote radioresistance in rectal cancer by facilitating DNA damage repair**

Yue Yu^1#^, Tingting Liu^2#^, Guanyu Yu^1#^, Hang Wang^2^, Zhipeng Du^1,3^, Yuanyuan Chen^2^, Nan Yang^4^, Kun Cao^2^, Chunlei Liu^5^, Zhijie Wan^2^, Hui Shen^2^, Fu Gao^2*,^ Yanyong Yang^2^*, Wei Zhang^1^*

^#^Authors contributed equally to this work.

1 Department of Colorectal Surgery, Changhai Hospital, Naval Medical University, Shanghai, China;

2 Department of Radiation Medicine, Faculty of Naval Medicine, Naval Medical University, Shanghai, China;

3 School of Public Health and Management, Wenzhou Medical University, University Town, Wenzhou, Zhejiang, China;

4 Pharmacy Department, Qingdao Special Servicemen Recuperation Center of CPLA Navy, Qingdao 266071, China;

5 Chifeng Municipal Hospital, Chifeng Clinical Medical School of Inner Mongolia Medical University, Chifeng 024000, China;


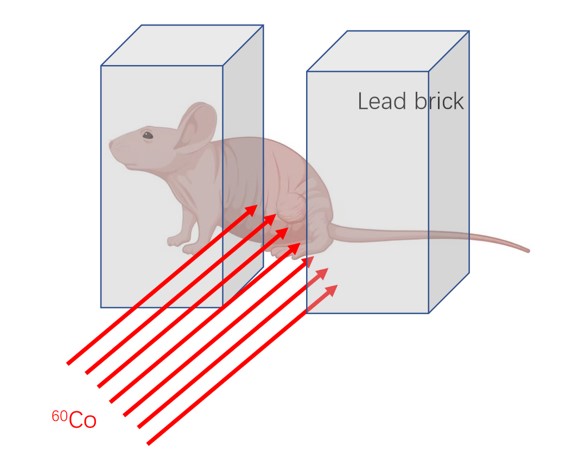


**Supplementary Fig. 1** Schematics of local irradiation strategy for tumor bearing mice of CDX and PDX model.


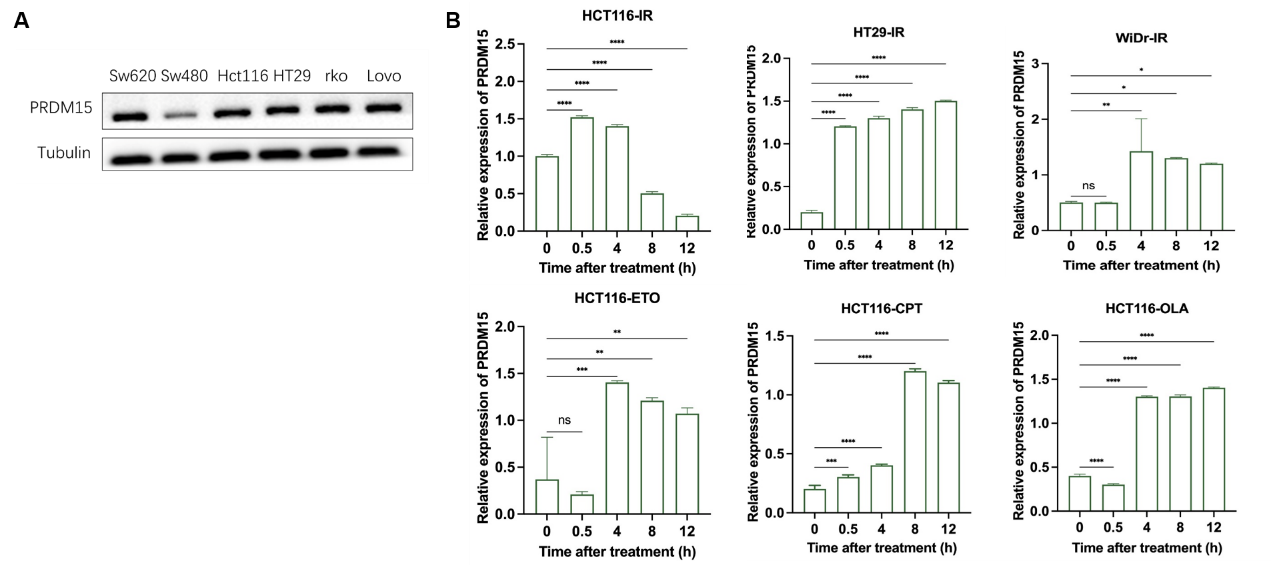


**Supplementary Fig. 2** (A) Expression of PRDM15 in different CRC cell lines. (B) Quantitation of western blots in Figure 1A and 1B. Error bars represent the SD of the mean of 3 independent experiments, two tailed Student’s t test was performed to compare the expressions at different time points with at 0h respectively, after analyzed with ANOVA. *P<0.05, **P<0.01, ***P<0.001 and ****P<0.0001 versus negative control.


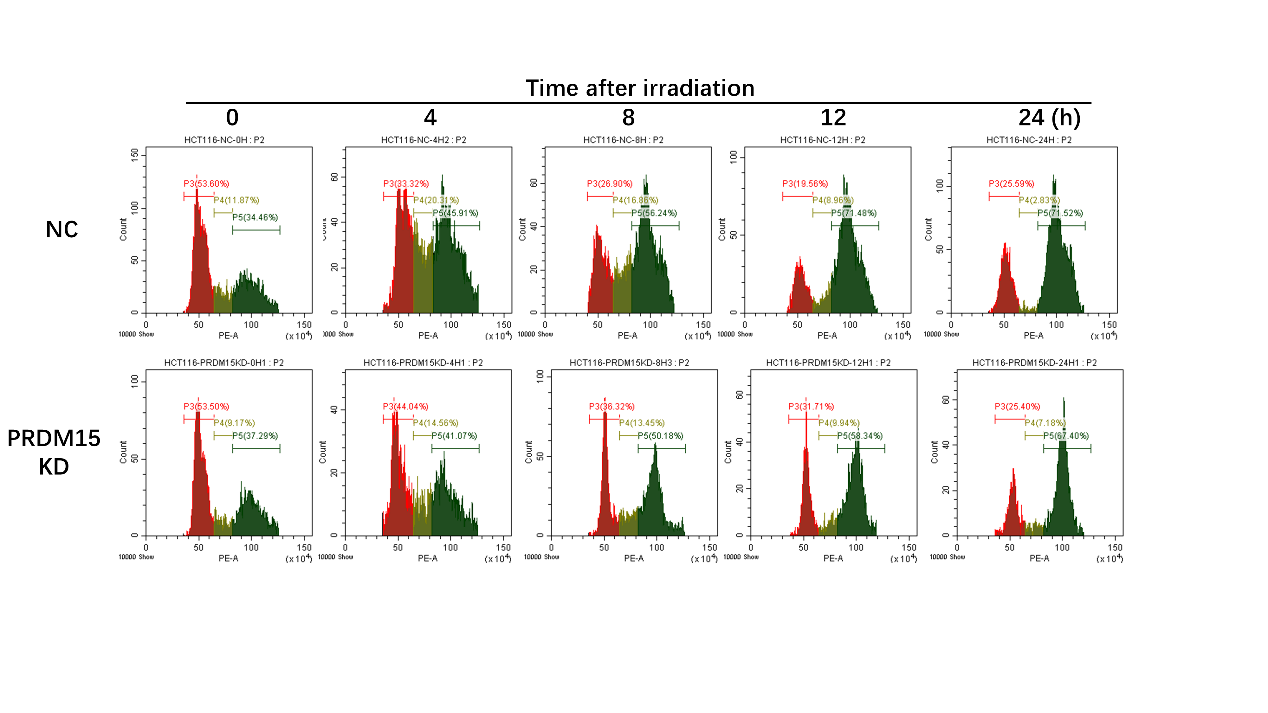


**Supplementary Fig. 3** Flow cytometry analyzing cell cycle distribution in response to IR (Relative to Fig. 2I).


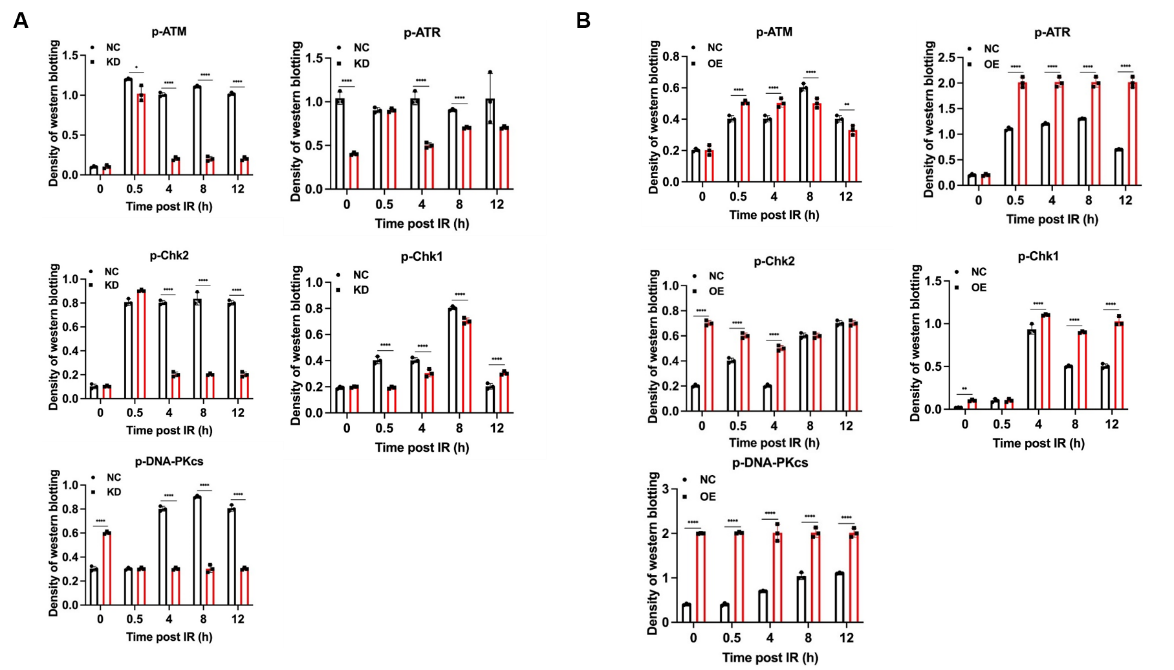


**Supplementary Fig. 4 Quantitative analysis of raw density of Western blot of molecules in DNA damage response**. (A) Quantitative analysis of raw density of Western blot in PRDM15-NC and PRDM15-KD cells after IR at different time points. (B) Quantitative analysis of raw density of Western blot in PRDM15-NC and PRDM15-OE cells after IR at different time points. n=3 for each group per time point. Values are given as mean±SD. *P<0.05, **P<0.01, ***<0.001 and ****P<0.0001 versus negative control shNC group.


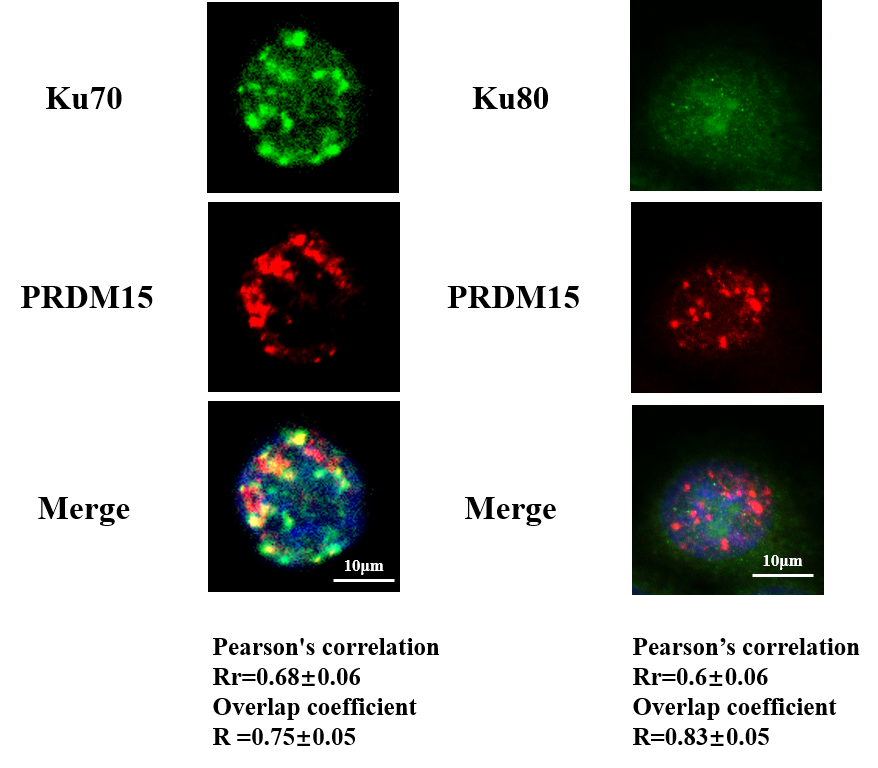


**Supplementary Fig.5 Immunofluorescent quantitated confocal pictures of the co-localization of PRDM15 with Ku70 and Ku80.** HCT116 cells at 0.5h after irradiation were used for immunofluorescence staining. Confocal microscopy was applied to capture co-localization, and Image J was used to analyze the statistical significance of co-localization. Pearson correlation coefficient of 0.5-1 is considered as co-localization.

**
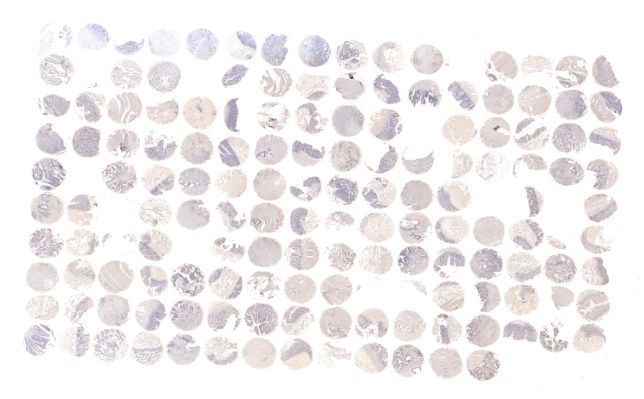
**

**Supplementary Fig. 6 Scanned image of tissues microarray including CRC patients classified with radiosensitivity by TRG score.**

**Supplementary Tables**

**Supplementary Table 1**

**Supplementary Table 2**

**Supplementary Table 3**

**Supplementary Table 4**

| **Table S4. The H-score of each sample in the tissue microarray** | | |
| --- | --- | --- |
| **Number** | **TRG** | **H score** |
| A01 | 2 | 82.154 |
| A02 | 2 | 34.808 |
| A03 | 0 | 15.498 |
| A04 | 0 | 25.851 |
| A05 | 3 | 14.889 |
| A06 | 3 | 9.908 |
| A07 | not found | 7.668 |
| A08 | not found | 0 |
| A09 | 3 | 0 |
| A10 | 3 | 0.261 |
| A11 | 3 | 7.956 |
| A12 | 3 | unable to detect |
| A13 | 2 | 63.69 |
| A14 | 2 | 164.948 |
| A15 | 3 | 252.745 |
| A16 | 3 | unable to detect |
| B01 | 3 | 174.479 |
| B02 | 3 | unable to detect |
| B03 | 0 | 83.18 |
| B04 | 0 | 68.266 |
| B05 | 3 | unable to detect |
| B06 | 3 | 20 |
| B07 | 2 | 98.689 |
| B08 | 2 | 67.114 |
| B09 | 3 | 34.088 |
| B10 | 3 | 10.217 |
| B11 | 2 | 47.695 |
| B12 | 2 | 38.652 |
| B13 | 3 | 111.439 |
| B14 | 3 | 165.126 |
| B15 | 3 | 119.4 |
| B16 | 3 | 75.332 |
| C01 | 3 | 71.005 |
| C02 | 3 | 70.136 |
| C03 | 1 | 107.326 |
| C04 | 1 | 87.451 |
| C05 | 3 | 132.919 |
| C06 | 3 | 30.515 |
| C07 | 3 | 87.859 |
| C08 | 3 | 145.1 |
| C09 | 3 | 76.586 |
| C10 | 3 | 52.505 |
| C11 | 3 | unable to detect |
| C12 | 3 | 90.783 |
| C13 | 1 | 139.452 |
| C14 | 1 | 198.601 |
| C15 | 0 | 151.356 |
| C16 | 0 | 83.96 |
| D01 | 1 | 50.807 |
| D02 | 1 | 69.296 |
| D03 | 3 | 123.216 |
| D04 | 3 | 157.572 |
| D05 | 3 | 137.327 |
| D06 | 3 | 79.72 |
| D07 | 2 | 97.799 |
| D08 | 2 | 105.325 |
| D09 | not found | 202.705 |
| D10 | not found | 114.981 |
| D11 | 1 | 110.663 |
| D12 | 1 | 117.235 |
| D13 | 2 | 21.714 |
| D14 | 2 | 219.198 |
| D15 | 1 | 167.663 |
| D16 | 1 | 116.983 |
| E01 | 1 | 73.281 |
| E02 | 1 | unable to detect |
| E03 | 2 | 235.605 |
| E04 | 2 | 149.498 |
| E05 | 2 | 154.597 |
| E06 | 2 | 202.062 |
| E07 | 2 | 135.561 |
| E08 | 2 | 111.147 |
| E09 | 1 | 126.462 |
| E10 | 1 | 139.881 |
| E11 | 2 | 186.379 |
| E12 | 2 | 109.326 |
| E13 | 0 | unable to detect |
| E14 | 0 | 132.97 |
| E15 | 1 | unable to detect |
| E16 | 1 | 95.76 |
| F01 | 2 | 106.97 |
| F02 | 2 | 141.565 |
| F03 | 1 | unable to detect |
| F04 | 1 | 183.217 |
| F05 | 0 | 84.731 |
| F06 | 0 | 142.763 |
| F07 | 3 | 181.47 |
| F08 | 3 | 140.791 |
| F09 | 1 | 165.674 |
| F10 | 1 | 192.651 |
| F11 | 2 | 207.063 |
| F12 | 2 | 162.073 |
| F13 | 2 | 152.136 |
| F14 | 2 | 228.397 |
| F15 | 1 | 219.139 |
| F16 | 1 | 194.7 |
| G01 | 2 | 183.929 |
| G02 | 2 | 192.297 |
| G03 | 1 | 201.304 |
| G04 | 1 | 153.499 |
| G05 | 2 | unable to detect |
| G06 | 2 | 106.257 |
| G07 | 1 | 103.25 |
| G08 | 1 | 146.544 |
| G09 | 2 | 166.053 |
| G10 | 2 | 157.746 |
| G11 | 2 | 146.118 |
| G12 | 2 | 130.877 |
| G13 | 2 | 184.044 |
| G14 | 2 | 157.177 |
| G15 | 3 | 235.514 |
| G16 | 3 | 167.368 |
| H01 | 2 | 178.451 |
| H02 | 2 | 174.938 |
| H03 | 2 | 116.28 |
| H04 | 2 | 92.409 |
| H05 | 3 | 120.84 |
| H06 | 3 | 107.785 |
| H07 | 2 | 158.254 |
| H08 | 2 | 136.23 |
| H09 | 2 | 133.801 |
| H10 | 2 | unable to detect |
| H11 | 0 | unable to detect |
| H12 | 0 | 179.762 |
| H13 | 1 | 138.589 |
| H14 | 1 | 196.638 |
| H15 | 0 | 242.857 |
| H16 | 0 | 221.931 |
| I01 | 2 | 200.309 |
| I02 | 2 | 192.551 |
| I03 | 2 | 95.365 |
| I04 | 2 | 71.869 |
| I05 | 2 | 115.003 |
| I06 | 2 | 136.151 |
| I07 | 2 | 138.39 |
| I08 | 2 | 149.162 |
| I09 | 3 | 166.192 |
| I10 | 3 | 126.299 |
| I11 | 1 | 109.224 |
| I12 | 1 | 105.473 |
| I13 | 3 | 155.664 |
| I14 | 3 | 111.037 |
| I15 | 3 | 236.162 |
| I16 | 3 | 210.47 |
| J01 | 0 | 192.011 |
| J02 | 0 | 170 |
| J03 | 2 | 218.595 |
| J04 | 2 | 154.693 |
| J05 | 2 | 104.443 |
| J06 | 2 | 139.091 |
| J07 | 3 | 103.983 |
| J08 | 3 | 127.55 |
| J09 | 1 | 167.763 |
| J10 | 1 | 136.751 |
| J11 | not found | 153.377 |
| J12 | not found | 106.075 |
| J13 | 2 | 151.035 |
