# Supplementary material for: PRDM15 interacts with DNA-PK-Ku complex to promote radioresistance in rectal cancer by facilitating DNA damage repair
Source: Cell Death Dis. 2022 Nov 19;13(11):978. doi: 10.1038/s41419-022-05402-7 (PMC9675803; doi:10.1038/s41419-022-05402-7)
Supplement: Supplementary file 2 — Original Data File [file 41419_2022_5402_MOESM2_ESM.pdf]

Figure 1A

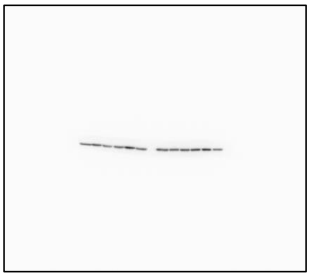

1A-gap-ht29-IR

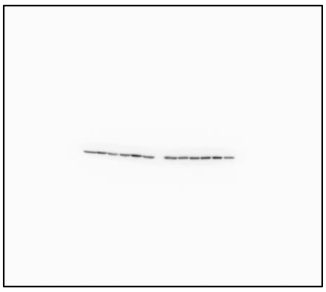

1A-HCT116-GAPDH

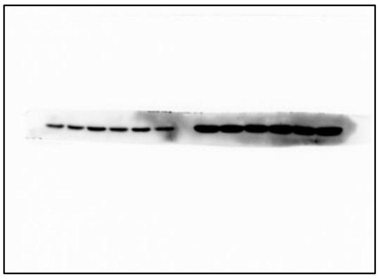

1A-gap-WiDr-IR

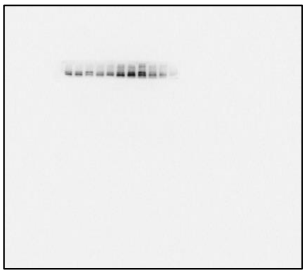

1A-HCT116-PRDM15

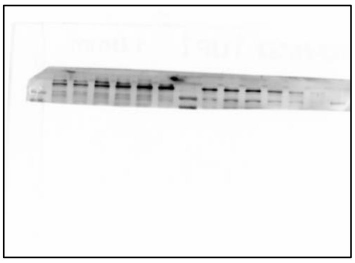

1A-HT29-PRDM15

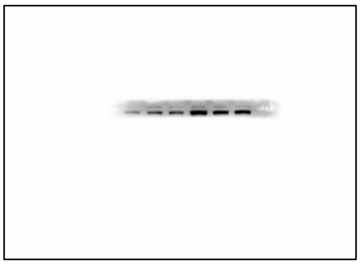

1A-WiDr-PRDM15

Figure 1B

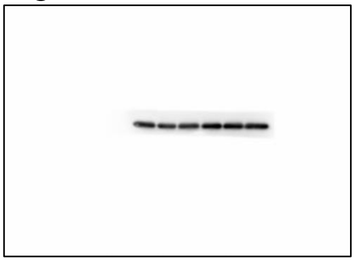

1B-gap-hct116-CPT

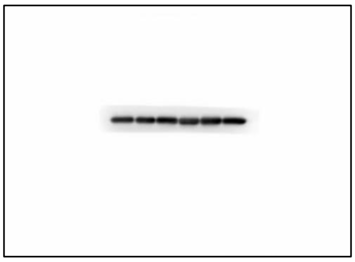

1B-gap-hct116-ETO

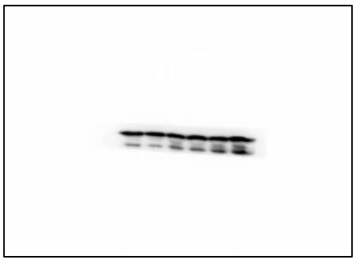

1B-gap-hct116-OLA

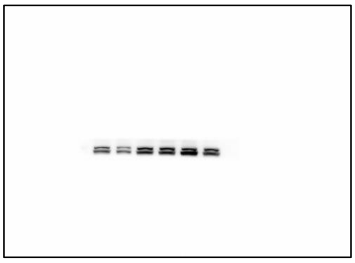

1B-OLA-PRDM15

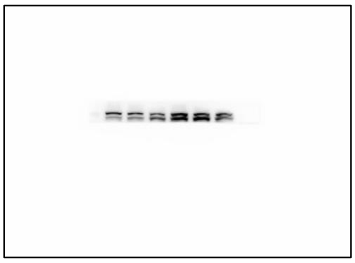

1B-CPT-PRDM15

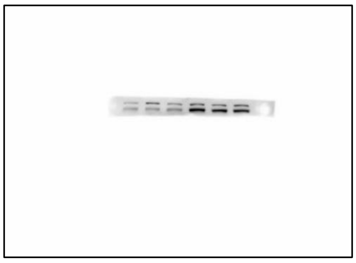

1B-ETO-PRDM15

Figure 1E

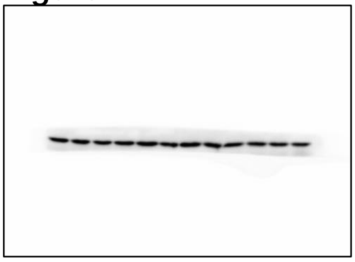

1E GAPDH

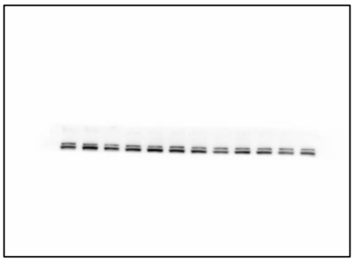

1E PRDM15

Figure 2A

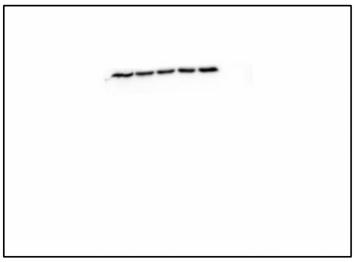

2A GAPDH

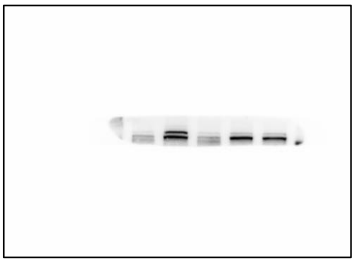

2A PRDM15

Figure 2B

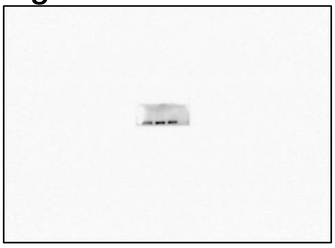

2B PRDM15

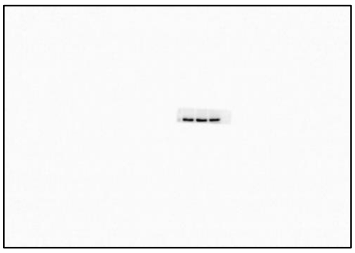

2B GAPDH

Figure 4A

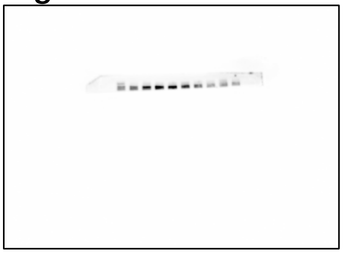

4A pDNA-PKcs

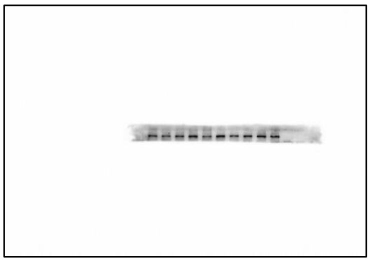

4A DNA-PKcs

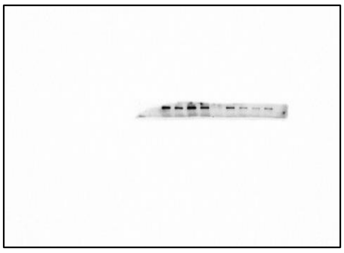

4A pATM

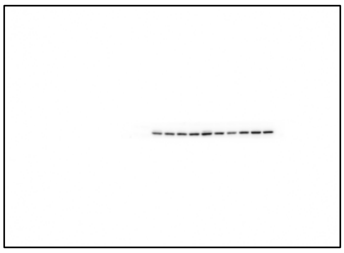

4A ATM

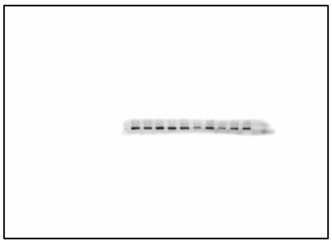

4A pATR

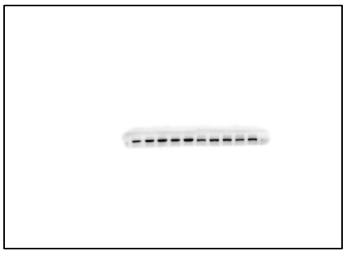

4A ATR

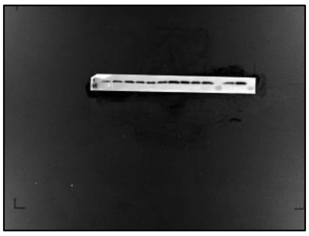

4A γ-H2AX

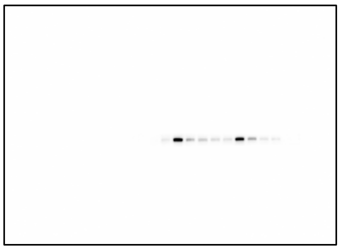

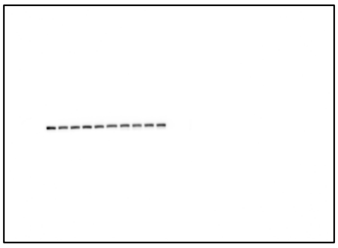

4A pKAP1

4A KAP1

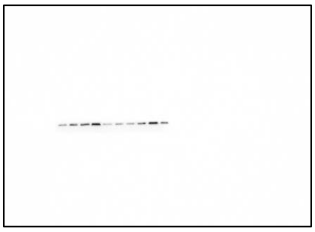

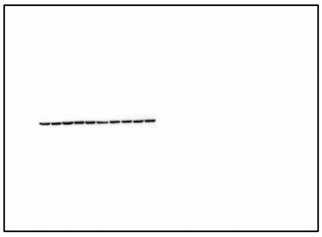

4A pchk1

4A chk1

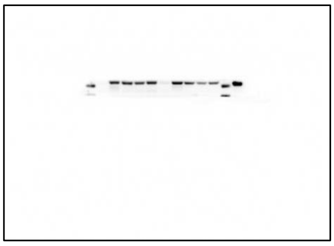

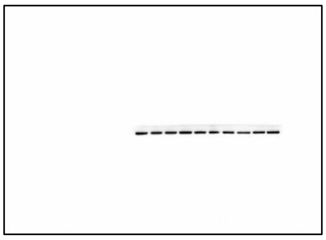

4A pchk2

4A chk2

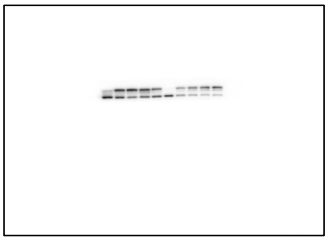

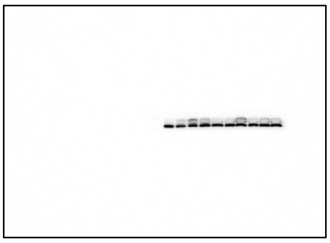

4A pRPA2

4A RPA2

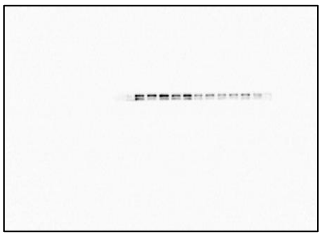

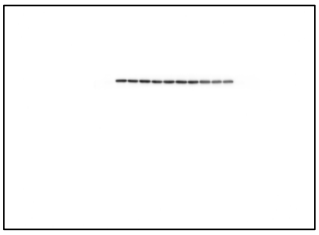

4A PRDM15

4A GAPDH

Figure 4B

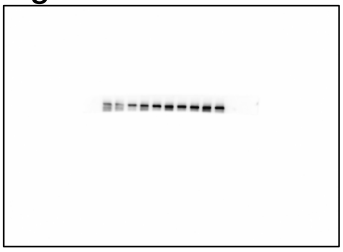

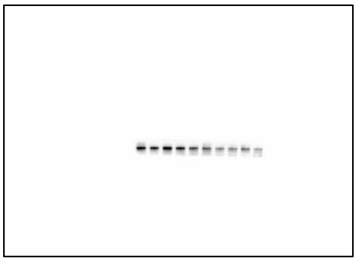

4B pDNA-PKcs

4B DNA-PKcs

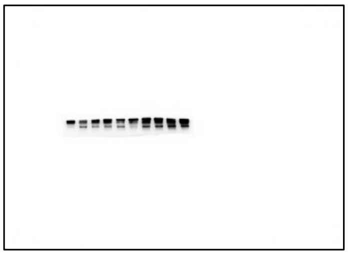

4B pATM

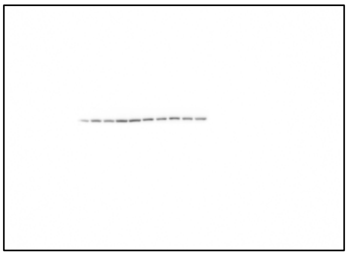

4B ATM

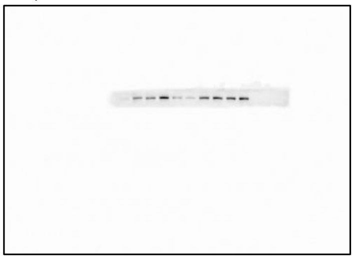

4B pATR

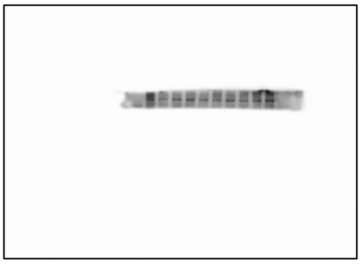

4B ATR

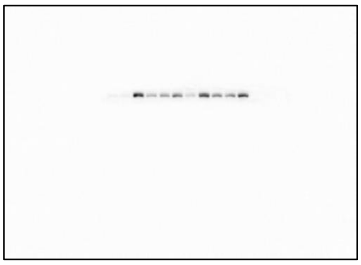

4B pKAP1

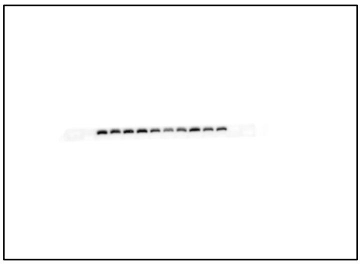

4B KAP1

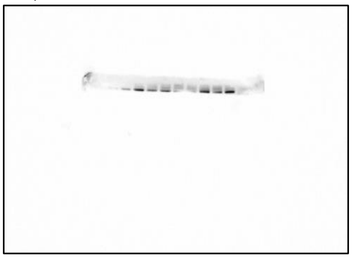

4B pchk1

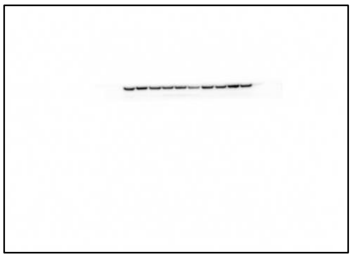

4B chk1

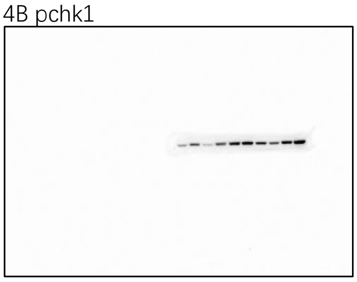

4B pchk2

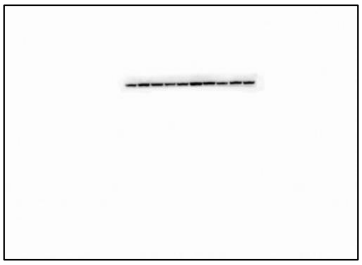

4B chk2

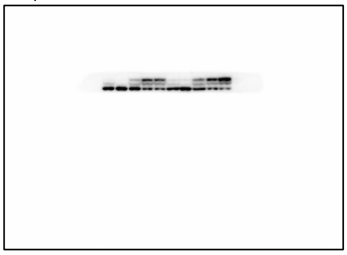

4B pRPA2

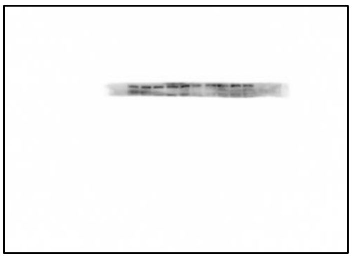

4B RPA2

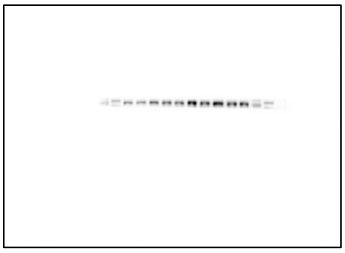

4B PRDM15

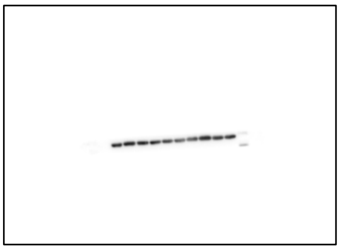

4B GAPDH

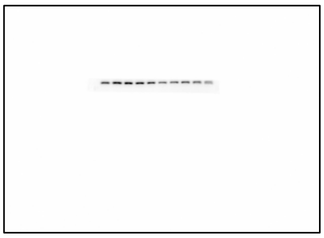

4B γ-H2AX

Figure 5A

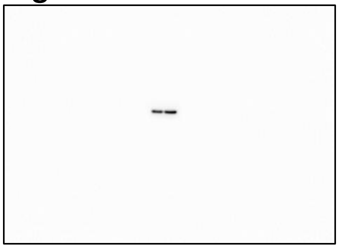

5A input GAPDH

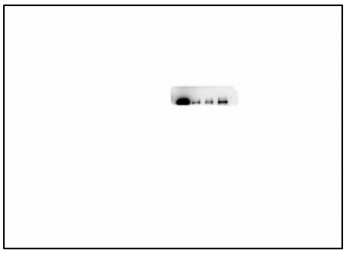

5A input-DNA-PKcs

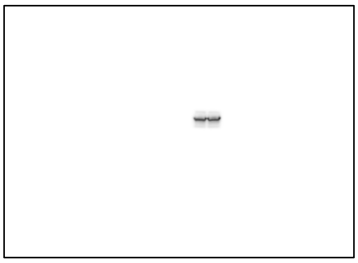

5A input Ku70

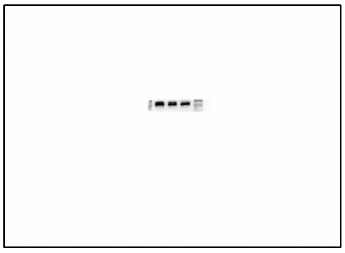

5A input-PRDM15

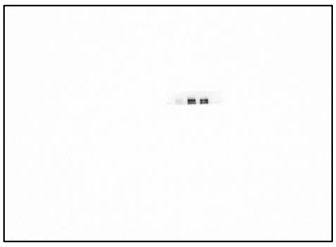

5A input-KU80

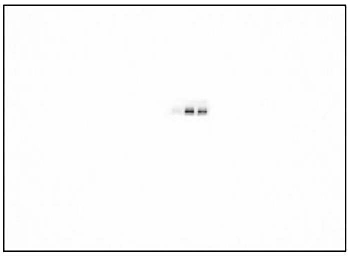

5A IP-PRDM15

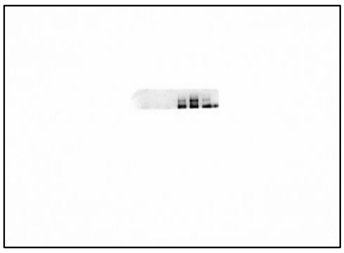

5A ip DNA-PKcs

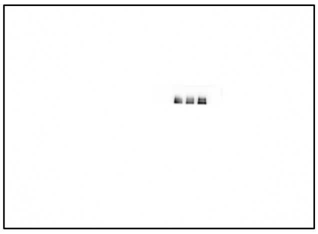

5A ip-KU70

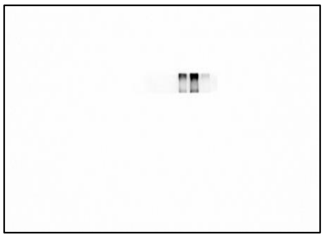

5A ip-KU80

Figure 5B

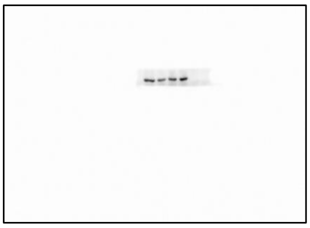

5B input GAPDH

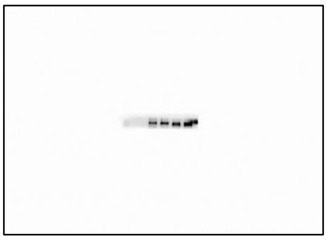

5B input-DNA-PKcs

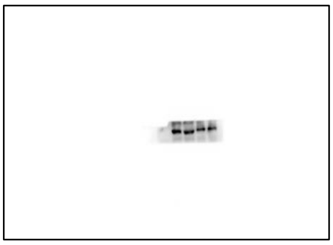

5B input Ku70

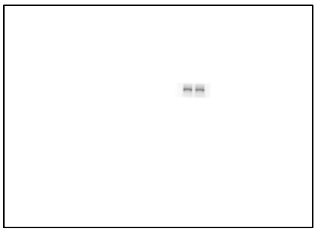

5B input-PRDM15

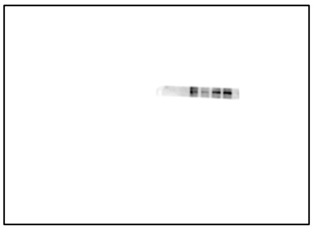

5B input-KU80

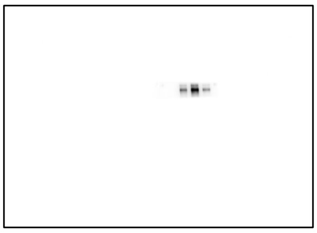

5B IP-PRDM15

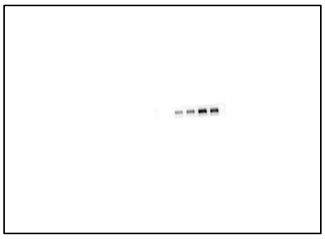

5B ip DNA-PKcs

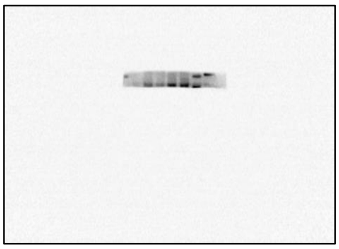

5B ip-KU70

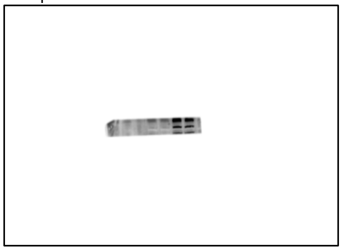

5B ip Ku80
